# Notes and News

**Published:** 1892-06-11

**Authors:** 


### Notes and News.

Collected and Communicated.

The Right Hon. Lord Sandhurst has consented to preside at the eighth annual meeting of the Hospitals Association, to be held at Charing Cross Hospital on Saturday, June 18th, at five p.m. It is hoped that many hospital authorities will make a point of being present, under the special circumstances, to welcome Lord Sandhurst.

Ingenuity in the matter of charity appeals must provoke our admiration. The field has been so thoroughly and variously worked in all directions. A fresh possibility of securing attention has presented itself, and of this the rector of Whitechapel has had the wisdom to make use of. We received a letter-card recently, which we opened with care, and discovered therein an appeal for funds. We could not pass it over, and the idea struck us as an excellent one, which we hasten to pass on.

Is cheese digestible? Dr. Kleuze has recently answered this question by a most uncompromising negative. Various kinds of cheeses were artificially digested with gastric juice, and under the most favourable circumstances they took very nearly twice as long as the ordinary foods contained in a mixed dietary. The reason for this is probably the fact that, although cheese for the most part consists of casein, a highly digestible substance, it is so intimately mixed with various kinds of fats, which are not acted upon by gastric juice, that the gastric juice is separated, as it were, from the digestible casein by an indigestible envelope of fat. Therefore, if large pieces of cheese are swallowed, they can neither be digested by the stomach, nor are they passed on to the intestines, there to be digested by the intestinal juices, but they remain in the stomach and irritate it to such an extent that the symptoms of indigestion supervene, and *hinc illæ lachrymæ*. Our advice is, therefore, not to exclude cheese from the household dietary, but rather to be careful to eat it in small pieces and masticate it carefully in the mouth, mixing it as thoroughly as possible with bread or some other food substance, as mastication of cheese by itself is very difficult, owing to its tenacious consistence.

In the new pharmacopæia in the United States, now in course of preparation, the metric weights and measures will be adopted throughout, to the entire exclusion of the English weights and measures hitherto used. It is considered by many that this is but the thin end of the wedge, and that sooner or later the metric weights and measures will be adopted for all purposes in the United States. In all the States of South America this system and none other is in use, and there is a growing feeling that an international system of weights and measures will do much to simplify and develop the commercial intercourse of the nations in question, as well as those who have adopted the metric system in Europe.

Those who work in these days of high pressure, and those who watch the effect of arduous work, especially amidst unfavourable surroundings, can testify to the positive necessity of change and rest if the workers' efficiency is to be maintained. Convalescent Homes, valuable as they are, do not reach such cases, and yet the needs of tired workers are very great. Miss Charlotte Mason, in the homes established by her now twenty years ago, has done her best to provide for the wants of women workers in missionary efforts amongst the poor or in foreign parts. One of her Houses of Rest is in Finchley Road, and the other is at Eastbourne. During the past year 800 inmates have passed through the homes. Visitors usually make a stay of one month, and it is difficult to over-estimate the benefit they derive, of which they themselves give grateful testimony. The payments received of visitors do not in any way cover expenses, which are met by voluntary subscriptions, and from time to time the work has met with very generous friends. Nevertheless, fresh subscriptions are much needed at the present time. It would appear that a personal visit to the homes produces undoubted approval to judge of the sums collected by means of the hall-box. In the event of a sufficient sum being collected, it is proposed to establish an extension by way of a Home for Aged Workers. A special donation fund is opened for this purpose.

Organisations for providing town children with holidays in the country has long flourished, but up to 1888 no provision was made for sending women and girls into the country for a little rest and relaxation. Many men belong to associations which, through the medium of annual fêtes, provide their members with opportunities of enjoying outdoor recreation, and they are besides enabled to take independent excursions of some days in a way that women are debarred from doing. The Factory Girls' Holiday Fund, of which the Princess Mary of Teck is Patron, is a most beneficial institution. Those who have watched the results accruing from it report on the good moral physical effect upon the girls. In many cases the girls try and save towards paying for the holiday themselves, but of course such subscriptions are merely nominal, the girls being actually the breadwinners of the family very frequently, and their earnings exceedingly small. The girls stay a week or a fortnight in some locality chosen by the Association, and under charge of some of the workers. The reports on their conduct, on the whole, is very gratifying. Those who should happen to have visited the scenes of these girls' labours, and their often wretched homes, cannot hesitate to encourage so excellent an effort in the direction of physical and moral improvement, and added happiness and brightness for these factory girls.

